# Antidiabetic Potential of Monoterpenes: A Case of Small Molecules Punching above Their Weight

**DOI:** 10.3390/ijms19010004

**Published:** 2017-12-21

**Authors:** Solomon Habtemariam

**Affiliations:** Pharmacognosy Research Laboratories & Herbal Analysis Services, University of Greenwich, Central Avenue, Chatham-Maritime, Kent ME4 4TB, UK; s.habtemariam@herbalanalysis.co.uk; Tel.: +44-208-331-8302 or +44-208-331-8424

**Keywords:** monoterpenes, diabetes, obesity, multiple mechanisms

## Abstract

Monoterpenes belong to the terpenoids class of natural products and are bio-synthesized through the mevalonic acid pathway. Their small molecular weight coupled with high non-polar nature make them the most abundant components of essential oils which are often considered to have some general antioxidant and antimicrobial effects at fairly high concentrations. These compounds are however reported to have antidiabetic effects in recent years. Thanks to the ingenious biosynthetic machinery of nature, they also display a fair degree of structural complexity/diversity for further consideration in structure-activity studies. In the present communication, the merit of monoterpenes as antidiabetic agents is scrutinized by assessing recent in vitro and in vivo studies reported in the scientific literature. Both the aglycones and glycosides of these compounds of rather small structural size appear to display antidiabetic along with antiobesity and lipid lowering effects. The diversity of these effects vis-à-vis their structures and mechanisms of actions are discussed. Some key pharmacological targets include the insulin signaling pathways and/or the associated PI3K-AKT (protein kinase B), peroxisome proliferator activated receptor-γ (PPARγ), glucose transporter-4 (GLUT4) and adenosine monophosphate-activated protein kinase (AMPK) pathways; proinflammatory cytokines and the NF-κB pathway; glycogenolysis and gluconeogenesis in the liver; glucagon-like-1 receptor (GLP-1R); among others.

## 1. Introduction

Several estimates on the current level of diabetes and its projected cases in the next few decades have been made in recent years. According to the World Health Organization’s (WHO), there were 422 million cases in 2014 with 8.5% prevalence that rose from 4.7% in 1980 [1]. Similarly, the major risk factor of diabetes has been recognized as obesity which prevalence in 2014 was 600 million; while the number of adults (18 years and older) reported as overweight were more than 1.9 billion [2]. As we are living in an era where more of the world population are suffering from consequences of excess calories than undernutrition, the cost of diabetes and obesity will remain the major social burden to societies in the next few generations. To date, diabetes is already a direct cause of death for millions of people annually but its impact is even more severe in its association with other diseases such as cardiovascular complications, organ failure (e.g., kidney) and disabilities, such as blindness and limb amputations [1].

There is as yet no drug of cure for diabetes and the current therapeutic approaches are directed on the management of the disease, primarily on glycemic control. The disease itself, also called diabetes mellitus, is manifested when there is persistent hyperglycemia in the blood arising from either insufficient (or not at all) amount of insulin released from the pancreatic β-cells and/or resistance to insulin is developed by vital organs. The most uncomplicated diabetes case is type-1 diabetes (T1D) were pancreatic β-cells are destroyed through an autoimmune-mediated reaction. On the other hand, type-2 diabetes (T2D) is characterized by insulin resistance with major risk factors being age and obesity. The disease is complex and may involve impaired insulin secretion and β cell death along with various metabolic dysregulations.

Since its discovery in the 1920’s, insulin has been taking the center stage in the therapy of both T1D and T2D [3,4]. Other classes of antidiabetic drugs include the α-glucosidase inhibitors (e.g., acarbose, miglitol and voglibose) that target carbohydrate digestion in the gut thereby limiting the availability of glucose taken up by the blood; the biguanides (the metformin-like compounds) that suppress glucose release/production in the liver; the thiazolidinediones such as glitazones which increase the sensitivity of insulin target organs; the insulin secretagogue sulfonylureas and the meglitinides; the glucose-dependent insulinotropic polypeptides (GLP-1) analogues; the DDP-4 inhibitors, among others [5]. These drugs have numerous side effects including gastrointestinal complications and loss of efficacy after pronged usage [6,7]. The high cost and patient compliance have also been other issues in diabetes therapy considering the required long period of treatment and repeated dosage. Hence, the urgency of identifying novel antidiabetic drugs with safer, more efficacious and cheaper profile cannot be overemphasized. Given diabetes being a complex disease, the rational for the search of multifunctional compounds that target multiple mechanisms of the diffused pathology has been advocated [8]. Furthermore, the antidiabetic potential of many natural products especially polyphenols that combine antioxidant, antiinflammatory, enzyme inhibition and various other general and specific insulin signaling modulatory effects have been outlined in recent years [9,10,11]. In this review, attention is given to structurally the smallest molecular weight group of compounds of the terpenoids classes of natural products, monoterpenes. Attempt is also made to scrutinize their antidiabetic activity profile built from their in vitro and in vivo data. As antidiabetic agents, monoterpenes now, somehow unexpectedly, appears to be recognized as small molecules punching a lot more than their weight.

## 2. Chemistry

**Figure 1:**
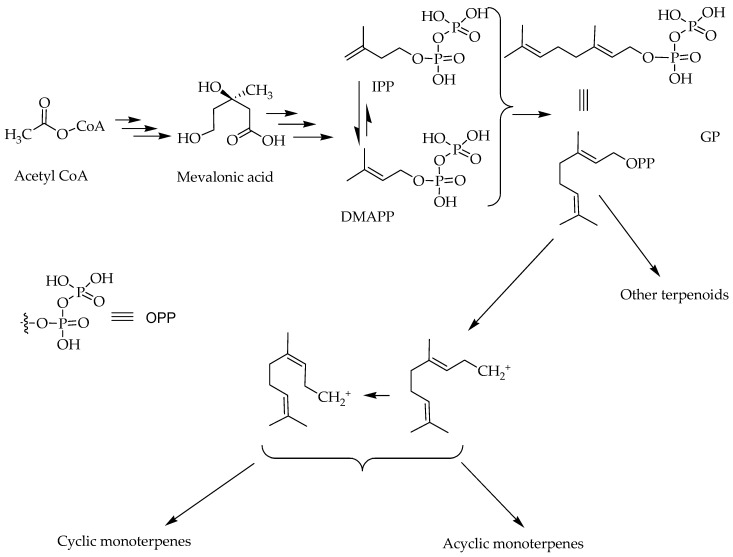
An overview of biosynthesis pathway of monoterpenes; isopentenyl diphosphate (IPP) and dimethylallyl diphosphate (DMAPP); glycogen phosphorylase (GP); OPP: diphosphate leaving group.

Like all other terpenoids, the monoterpenes are constructed from repeating units of basic 5-carbon building blocks called isoprenes [12,13]. Their biosynthesis start from the simplest primary metabolite acetyl-CoA that goes through a series of biosynthetic pathways involving the mevalonic acid. Accordingly, terpenoids are often called products of the mevalonic acid pathway of secondary metabolites [14,15,16]. The conversion of the primary metabolites into the secondary, terpenoids, natural products involve the synthesis of the key 5-carbon reactive intermediates, isopentenyl diphosphate (IPP) and dimethylallyl diphosphate (DMAPP). The condensation of these two isoprene units gives rise to a 10-carbon skeleton geranyl diphosphate (GPP): the immediate precursor of all monoterpenes (Figure 1). Sequential addition of further isoprene units to GPP leads to sesqueterpenes (15 carbon) and diterpenes (20 carbon) or their dimers, triterpenes (30 carbon) and tetraterpenes (40 carbon) respectively. The two 5-carbon terpenoid skeletons (IPP and DMAPP) could themselves give rise to a handful of 5 carbon derivatives that exist in alcohol, acid or hydrocarbon forms but they mostly incorporate into other secondary metabolites as ether or ester derivatives. Hence, the smallest but most structurally diverse group of terpenoids are represented by the monoterpenes.

**Figure 2:**
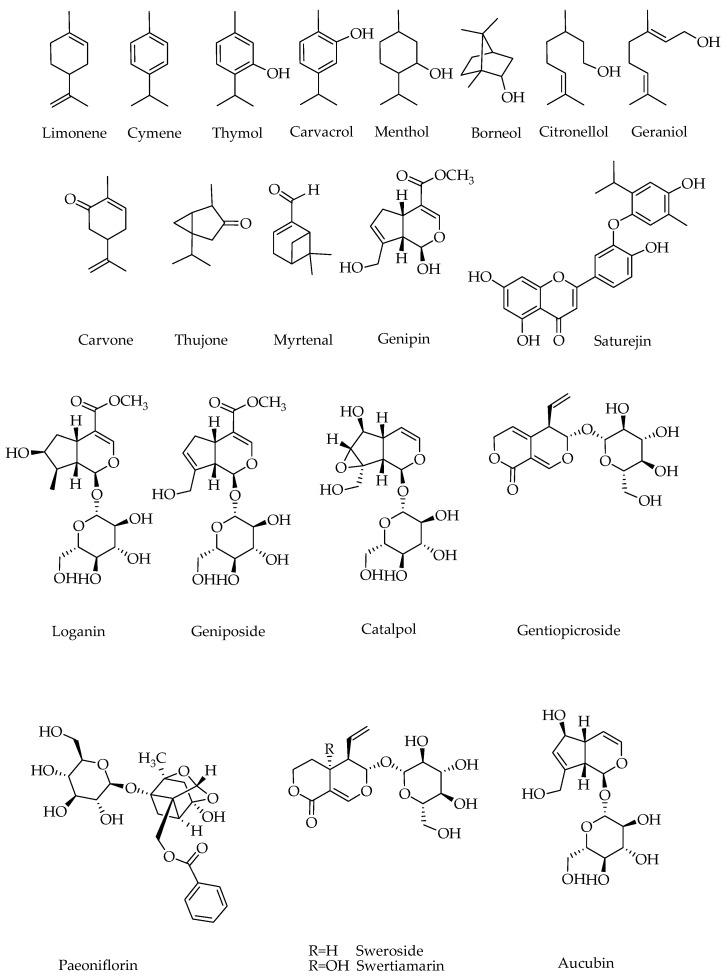
Structures of monoterpenes discussed in this review as antidiabetic agents.

Despite their small atomic mass (10 carbon), the monoterpenes have remarkable structural diversity owing to the diphosphate leaving group of the GPP. The resulting unstable cations (Figure 1) undergoes a series of reactions including addition reaction typically leading to incorporation of a hydroxyl group, double bond rearrangements and unsaturation that all eventually lead to stable structures of the monoterpenoid class (Figure 2). In fact, the hallmark of diversity in monoterpenoids that arose from just one precursor GPP (or its isomer neryl diphosphate) through a serious of enzyme catalyzed reactions including cyclization, hydroxylation, dehydrogenation, oxidation and/or reduction, isomerization, and conjugation have been the subject of over a century-old research for natural product chemists.

For most monoterpenoids, their small molecular weight coupled with their nonpolar nature means that they are easily extracted by either non-polar solvents (like hexane), supercritical CO_2_ or steam distillation. There are however monoterpenes mostly existing in glycosylated form and hence are very polar. The iridoids (Figure 2) are such group of compounds with *cis*-fused cyclopentano pyran ring system in their structure [16]. The monoterpenes can also be incorporated into other structures such as polyphenol (e.g., phenolic acids and flavonoids). In this review, biological activities that solely attributes to the monoterpenes are discussed.

## 3. General Function in Nature

Terpenoids play important roles in the organisms (bacteria, fungi, plants, and animals) that produce them. The large volume of volatile compounds that plants utilize to attract pollinator insects or deter herbivores belong to the monoterpene class of compounds. As such, the fragrances of herbs, spices, flowers and fruits are attributed to the essential oils which are dominated by monoterpenes. The various roles played by monoterpenes as signaling molecules in plant metabolism, plant-plant and plant-animal interactions have been extensively reviewed [12,17,18,19]. The monoterpenes are also among the best exploited group of compounds by mankind. The most prominent use once again relates to them being the major component of essential oils that make them valuable in the perfumery industry. Within the food industry, the flavoring and preservative potential of essential oils have been well understood along with their general antimicrobial and antioxidant effects. They are also exploited as pharmaceutical agents [19,20,21] and in agrochemical industries primarily as insecticides [22].

The general mechanism of action of monoterpenes such as their antimicrobial and cough therapy have been mainly associated to their volatile nature allowing them to freely move through space inclining biological membranes and interact with various biomolecules. Some of these actions have long been perceived as non-specific that lack drug-like selectivity to particular receptors. For example, the hydrophobicity of these compounds and essential oils in general have been shown to account for their disruptive effect on bacterial cellular structures (e.g., cell membrane) and hence leading to cell death [23]. There are also reports on non-specific and non-competitive mechanism of action for the muscle relaxant effects of some monoterpenes. For example, Boskabady and Jandaghi [24] have shown that the relaxant effect of carvacrol on tracheal smooth muscles of guinea pigs could not be accounted by effects via β2-adrenergic stimulatory, histamine H1, and muscarinic blocking. On the other hand, there are reports on specific effects of monoterpenes on receptors, such as in the cooling effect of menthol through action on thermoreceptors, even though the lack of specific mechanism for nasal decongestant action by monoterpenes is still the subject of intense discussion [25,26]. A non-competitive manor of receptor inhibition by monoterpenes have also been reported and exemplary findings include the direct interaction of linalool with the *N*-methyl-d-aspartate receptors [27] and nicotinic acetylcholine receptor inhibition by borneol [28]. Not surprisingly, the antidiabetic effects of monoterpenes were only coming to light in the last decade but now appear to gain lots of momentum. In the following sections, some exemplary effects collected from in vitro and in vivo studies are presented to highlight their promise in diabetes as well as insulin resistance and obesity.

## 4. Antidiabetic Potential of Monoterpenes

### 4.1. In Vitro Protective Effects

In vitro experiments (Table 1) on the antidiabetic effects of monoterpenes include cell culture studies using pancreatic β cells, muscles, adipocytes and liver cells among others; as well as enzyme/protein-based assays [29,30,31,32,33,34,35,36,37,38,39,40,41,42,43,44,45,46,47,48]. Of these, the study by Tan et al. [45] included direct comparison of activity of a number of commercially available monoterpenes: geraniol, nerol, citral, (*R*)-(−)-linalool, (*R*)-(+)-limonene, (*S*)-(−)-perillyl alcohol, (*R*)-(+)-β-citronellol, (*S*)-(−)-β-citronellol, α-terpineol, l-menthol, γ-terpinene and terpinolene. Their study employed in vitro antioxidant; α-amylase and α-glucosidase enzyme inhibition; and glucose uptake and lipid metabolism in 3T3-L1 adipocytes. Even though these compounds have been shown to have some radical scavenging properties (DPPH and ABTS radicals) at higher concentrations such as 10 and 100 mM, the observed activity is not of therapeutic relevance. In fact, considering their structure (Figure 1), that lack a phenolic structural moiety (with few exceptions), they are not expected to display potent antioxidant effect and/or direct radical scavenging. Over the years, we have shown the potent direct radical scavenging effects of polyphenolic compounds particularly those with catechol functional groups and those optimized with the flavonoids skeleton [11,49,50,51,52,53,54,55,56,57,58,59,60,61,62]. Hence, apart from compounds like carvacrol and thymol with phenolic skeleton, most monoterpenes (Figure 2) do not seem to have direct radical scavenging mechanism that account for their antidiabetic effects. With respect to inhibition of key carbohydrate enzymes (α-amylase and α-glucosidase) inhibition, these compounds were reported to act at 10 mM range [45] and is once again of no therapeutic relevance. In this connection, we have shown that many promising natural products such as flavonoids and polyphenols are acting at micromolar range [9,50,52,53,62,63,64,65]. On the other hand, the most prevalent effect of these monoterpenoid compounds were on glucose uptake and lipid metabolism in 3T3-L1 adipocytes cell culture [45]. (*S*)-(−)-β-citronellol, terpinolene and (*R*)-(−)-linalool did not affect the glucose uptake in 3T3-L1 adipocytes; while geraniol, citral, (*R*)-(+)-limonene, (*R*)-(+)-β-citronellol, nrerol, (*S*)-(−)-perillyl alcohol, γ-terpinene and α-terpineol showed some degree of activity (up to 21% inhibition) when tested at 1 µM. Readers should bear in mind that this concentration is so small and hence promising but a dose-dependent effect profile was not reported for these compounds. Similarly, the free glycerol released into the medium in the cell culture was measured, and at 1 µM concentrations, the lipolysis effect of (*R*)-(+)-limonene, (*S*)-(−)-perillyl alcohol, (*R*)-(+)-β-citronellol and geraniol were shown [45]. Once again, this is a screening result and does not show the concentration range and total profile of activity for these compounds. On the other hand, (*R*)-(+)-limonene treatment did not affect the mRNA expression of PPAR-γ in 3T3-L1 adipocytes; it increased the mRNA expression of GLUT1 by 1.2-fold whereas the mRNA expression of GLUT4 remained unchanged. These were all at a fixed concentration of one dose (1 µM).

The general antidiabetic effect of monoterpenes through specific biological targets are illustrated in Table 1. Even though essential oils are known for general antioxidant and enzyme inhibitory activities including α-glucosidases, such effect may not be therapeutically relevant as it occur at relatively high concentrations. There is a great deal of diversity among the monoterpenes however as some such as thymol are phenolic in nature while the vast majority are highly non-polar compounds (Figure 2). As discussed above, direct reactive oxygen species and radical scavenging (ROS) is mainly a function of polyphenolic compounds, which (with few exceptions such as thymol and carvacrol) are poorly represented in monoterpenes. Hence, such biological effect is not addressed here as the major mechanism of action of these compounds for their antidiabetic effects. It is interesting to note however that when monoterpenes are incorporated into other structural groups such as flavonoids as exemplified by saturejin, a far better direct antioxidant and enzyme inhibitory effects were obtained [46].

A number of other in vitro experiments have been carried out to assess the effect of monoterpenes on cultured pancreatic β cells. This has shown remarkable results as concentrations in micromolar range have been demonstrated to potentiate insulin secretion (Table 1). Various experiments on adipocytes, hepatocytes and muscle cells also confirmed that these compounds can effectively increase glucose uptake through upregulation of the glucose transporter (GLUT4) translocation. As discussed in the following sections, some key pharmacological targets at molecular level including the insulin signaling pathways have been implicated. Genipin and geniposide as well as iridoids and their glycosides have also been shown to display potential antidiabetic effects in vitro. In the cultured muscle myotube cells, an increase in the phosphorylation of insulin receptor substrate-1 (IRS-1) and other signal transduction pathways leading to increased intracellular calcium concentrations have been reported (Table 1). The AMPK pathway has also been shown to be involved in the protection of pancreatic β cells by geniposide [32,33]. Furthermore, activation of the glucagon-like-1 receptor has been implicated in the insulin secretion promotion by geniposide [34]. The role of PPARγ and phosphatidyl inositol 3-kinase (PI3K) are also shown to be involved in the effect of these compounds in neuronal cells. As discussed in the following texts, the PI3K involvement, which has been confirmed by using specific inhibitor (wortmannin), is a further interesting insight into the possible mechanism of action of monoterpenes [31]. Hence, at concentrations as low as 10 µM, these compounds can protect pancreatic β cells and other cells such as neurons; promote insulin secretion and facilitate glucose uptake. Moreover, amelioration of the pro-inflammatory cytokines (e.g., TNF) in adipocytes and lipid accumulation in liver cells have been shown to be induced by geniposide. The involvement of the GLP-1R signaling pathway in the antidiabetic effect of geniposide has also been confirmed by specific receptor antagonist, exendin [38,39]. The production of TNF and free fatty acids (FFAs) in adipocytes and macrophages in vitro could also be ameliorated by monoterpenes such as paeoniflorin [42,43], swertiamarin [47] and thujone [48].

### 4.2. In Vivo Antidiabetic Effects

The remarkable feature of monoterpenes is that they have shown promising antidiabetic effect in vivo despite their rather simplistic structural appearance [35,66,67,68,69,70,71,72,73,74,75,76,77,78,79,80,81,82,83,84,85,86,87,88,89,90,91,92,93,94,95,96,97,98]. The list of monoterpenes with good effects on in vivo antidiabetic models including in the streptozotocin (STZ)-induced diabetic, high fat diet (HFD) fed and spontaneously obese mouse models are shown in Table 2. Some of these compounds are presented as simple hydrocarbons such as limonene and cymene; ketone or hydroxy derivatives; aromatic or non-aromatic skeletons; or glycosides of iridoids (Figure 2). In all cases, a promising antidiabetic effect at doses as low as 5 mg/kg have been observed. The variability in dose regimen and duration of study in these studies, however, do not allow direct comparison of potency between the various structural groups of the monoterpenes. The following observation were however evident as a general antidiabetic agents.

As shown in Table 2, hyperglycemia is the primary criteria assessed in all studies and the indicated monoterpenes have shown promising effects in all studied models. In addition to lowering the blood glucose level, the common antidiabetic effect assessment involves the measurement of glycated hemoglobin, primarily HbA1c. This is also indicated for many compounds studied (Table 2). In the liver, one of the main antidiabetic drugs target, increasing the level of glycogen level has been reported for borneol [67], citronellol [78] and myrtenal [90,91]. Modulation of key hepatic enzymes of glucose metabolism such as glucose-6-phosphatase and fructose-1,6-bisphosphatase; decreased glucokinase and glucose-6-phosphate dehydrogenase activities, have been reported for carvacrol [69], carvone [73], citronellol [78], geniposide [84], myrtenal [91] and paeoniflorin [82,93]. The common markers of hepatic function aspartate aminotransferase (ASP), alanine aminotransferase (ALA), alkaline phosphatase (ALP), and gamma-glutamyl transpeptidase have also been routinely measured in diabetes and their raised status in diabetes have been suppressed by carvacrol [69] and carvone [73]. Other organ functions primarily the kidney (cymene and logonin) and the brain (geniposide, thymol and d-limonene) have also been reported (Table 2).

It is important to note the effect of the studied compounds on gross weight of the animals especially given administration of STZ-induced diabetes is associated with weight loss. For example, administration of borneol [67], carvacrol [68] and swertiamarin [94] have shown to reverse the body weight loss in diabetes. On the other hand, the body weight gain in HFD-obese model could be ameliorated by monoterpenes. Even though, catalpol had no effect at the dose of 100 mg/kg (although it improved fasting glucose and insulin levels) [75], geniposide [83], paeoniflorin [82,93] and thymol [97,98] were among the monoterpenes that showed good potential antiobesity effect. Geniposide also displayed antiobesity effect in spontaneously obese Type 2 diabetic Tsumura Suzuki Obese Diabetes (TSOD) mice model [40].

Given that STZ administration primarily targets pancreatic β cells leading to insulin depletion and hence diabetic condition, enhancing insulin secretion is one the well-defined mechanism of antidiabetic agents. While carvacrol did not have significant effect on the serum insulin level at the tested dose [72], carvone [73], citronellol [78], geniposide [82], geraniol [85], myrtenal [90] and swertiamarin [94] have all been shown to increase insulin level in the STZ-induced diabetes. As demonstrated for menthol [89] and myrtenal [91], improvement in the pancreatic and hepatic cellular and structures, as evidenced from histological studies, have been reported. On the other hand, in the HFD-obese model, hyperglycemia is associated with a rise in insulin level which appeared to be targeted/normalized by monoterpenes. Good examples are geniposide [84] and thymol [98].

As discussed above, antidiabetic effect is often measured by assessing the level of glycation of proteins including hemoglobin which has been shown to be suppressed by monoterpenes. Considering the link between glycation and oxidative stress, it is worth looking at the antioxidant effect of these compounds in vivo. Even though most monoterpenes are not known for their potent direct ROS scavenging effect in vitro (Table 1), their in vivo antidiabetic effect is associated with an improved antioxidant status (Table 2). For example, borneol [67] has been shown to enhance antioxidant status in diabetic animals including augmented level/activity of superoxide dismutase (SOD), catalase, reduced glutathione (GSH) in the liver and kidney, while concomitantly suppressing the level of oxidation marker, malondialdehyde (MDA). Similar results were obtained for aucubin [66], carvacrol [70,71], genipin [79], d-limonene [87], logonin [88], and thymol [96].

The antihyperlipidemic and antiobesity effect of monoterpenes appears to be well demonstrated in vivo. The diabetes-induced increase in the plasma or tissue levels of TC, TGs LDL-C, VLDL-C are often measured to assess the potential lipid lowering effect of drugs while raising the level of HDL is regarded as valuable parameter of lipid modulation. Borneol [67], carvacrol [72], genipin [79], geniposide [40], d-limonene [87], swertiamarin [94] and thymol [95,96] are classical examples of monoterpenes shown to display such effects (Table 2). The accumulation of fat in tissues as evidenced for geniposide and paeoniflorin have also been demonstrated along with the various effects in the HFD-induced diabetes (Table 2). Hand in hand with all these effects are the amelioration of the diabetes inflammation by various monoterpenes including effects on proinflammatory cytokines such as TNF along with the NF-κB pathway (Table 2). The various other effects of monoterpenes in animal models of diabetes including central effects and neuroprotection are listed in Table 2.

## 5. Bioavailability

Several studies on the absorption, distribution, metabolism, and excretion of monoterpenes have been published in recent years. Liu et al. [99] have studied the oral bioavailability of paeoniflorin in a single-pass “four-site” rat intestinal perfusion model and cultured Caco-2 cells. As expected, the absorption of this compound in cultured cells was slower than its aglycone (paeoniflorigenin). They have found that poor permeation, P-gp-mediated efflux, and hydrolysis via a glucosidase contributed to the poor bioavailability of paeoniflorin. When the crude plant extract preparation of *Paeonia lactiflora* roots (Chishao) that contain paeoniflorin as a major component (85.5%) was administered in human subjects by intravenous and multiple infusions, an elimination half-lives of 1.2–1.3 h was noted for paeoniflorin. After exposure to major organs, glomerular-filtration-based renal excretion has been found to be the major elimination pathway for this compound [100]. The bioavailability of paeoniflorin has been generally considered low in rabbit (7.24%) and rat (3.24%) after oral administration [101].

Oral administration of the monoterpene aglycones such as thymol and carvacrol has also been shown to result in their slow absorption into the blood stream [102]. Both unchanged and their glucuronide and sulfate conjugates have also been detected. For example, Dong et al. [103] have shown that carvacrol, as a substrate to uridine diphosphate-glucuronosyl transferase, can inhibit the enzyme. In another study of a clinical trial involving 12 healthy volunteers, no thymol could be detected in plasma or urine [104]. However, its metabolites thymol sulfate and thymol glucuronide were found in urine and the reported mean terminal elimination half-life was 10.2 h. Moreover, thymol sulfate was detectable up to 41 h after administration and urinary excretion normally followed over 24 h [104].

In a potential anticancer therapy study, administration of lemonene in women with newly diagnosed operable breast cancer indicated preferential concentration of the compound in the breast tissue, reaching high tissue concentration (mean of 41.3 μg/g tissue) while its major active circulating metabolite was reported to be perillic acid [105]. The accumulation of limonene on adipose tissues has also been described in other studies [106]. Oral administration of swertiamarin in rats has been shown to be associated with rapid and wide distribution in tissues with the highest amount obtained in the liver and kidney, perhaps indicating the possible metabolism and/or elimination sites [107]. As demonstrated for geniposide, administration of these compounds in the form of crude plant extract may be better for their bioavailability than the purified compounds [108].

There is no doubt that more data is needed to establish a clear bioavailability and/or pharmacokinetic profile of monoterpenes. The fact that these compounds display antidiabetic effect when administered orally and at even modest doses suggest that they can be absorbed/bioavailable to exert their action in the various organ systems. There distribution profile in tissues reported so far has also been promising although further studies on optimization and formulation studies are critical. The extreme nonpolar nature of the monoterpene aglycones imply that they can easily travel across cell membranes but their poor water solubility is a challenge, and given this fact, the observed antidiabetic effect should be considered remarkable. On the other hand, the glycosides of these compounds are also shown to display antidiabetic effects and may constitute another route for optimizing their antidiabetic effects.

## 6. General Summary and Discussion

The various in vitro and in vivo data now suggest that monoterpenes, despite their structural simplicity, have a promise to be seriously considered as antidiabetic lead compounds either by their own or as part of structural moieties in complex structures. Hence, the diverse mechanism of action reported in these studies deserve further scrutiny. A growing body of evidence show that the PPARγ play a vital role in the regulation of carbohydrate, lipid, and protein metabolism both in healthy and disease states (e.g., diabetes, obesity, metabolic syndrome, cardiovascular diseases, etc.). In the case of diabetes, the PPARγ with its predominant expression in adipose tissues (but also in macrophages and intestine) has profound effects in the regulation of adipogenesis, insulin sensitivity, and inflammation [109,110,111]. The role of PPARγ in key other insulin-target organs/tissues such as the liver and muscles has also been well understood. Even though the thiazolidinediones (TZDs) groups of antidiabetic drugs have been reported to have numerous undesirable side effects [112,113], targeting the PPARγ by receptor agonists remains an attractive pharmacological target for the treatment and prevention of metabolic disorders including diabetes. In this regard, the role of natural products as a source of potential drugs that lack the undesirable effects of thiazolidinediones have been advocated [114,115]. Hence, the modulatory effect of monoterpenes on the PPARγ appear to be a well-defined mechanism of antidiabetic action. Furthermore, monoterpenes such as carvacrol, thymol and others that showed antidiabetic effects are common ingredients of foods and flavors and hence not expected to display the undesirable effects of thiazolidinediones.

The role of the AMPK in regulating glucose metabolism and as a validated target for diabetes has gained lots of momentum in recent years. Drugs that activate the AMPK have been shown to enhance the phosphorylation of insulin receptor substrate 1 (IRS-1) Ser789 phosphorylation leading to the cascade of activation of phosphoinositide 3 kinase/protein kinase B (PI3K/PKB) signaling [116]. Such effects have been shown to be both insulin dependent (potentiation) and independent (in unstimulated state). The classical antidiabetic drug, metformin, also appear to act through activation of the AMPK pathway in various target organs [117]. Interestingly, the activation of AMPK in adipocytes has been shown to suppress lipogenesis while promoting energy dissipation suggesting potential antiobesity effect [118]. Even though some studies still show the paradoxical nature of the AMPK activation in glucose and lipid metabolism, mostly related to the level of stimulation (chronic verses acute) (e.g., [119,120]), the role of AMPK activators in various disease condition have been well documented [121]. In a recent review [122], the antidiabetic potential of natural products such as anthocyanins through effect on the AMPK and insulin-signaling pathways have been highlighted. Interestingly, monoterpenes have been shown to share this crucial mechanism of antidiabetic effect (Table 1).

The GLUT4 is an ATP-independent glucose transport system across cell membranes and predominantly expressed in adipose tissues and skeletal muscles. While under expression of the GLUT4 can predispose animals to diabetes and insulin resistance, its overexpression has been shown to overcome such physiological/pathological disorder [123,124]. The reduced level of GLUT4 has also been noted in muscles of diabetic patients. The coupling between insulin receptor activation in target organs with the known insulin action in GLUT4-mediated glucose uptake has been the subject of intense research in recent years. The insulin receptor substrate proteins phosphorylation has been shown to lead to the activation of PI3K which intern leads to the generation of phosphatidylinositol 3,4,5-trisphosphate (PIP3). The PIP3 in turn is linked to recruit protein kinase B (PKB), also known as Akt, that play a pivotal role in the insulin-dependent GLUT4 mobilization. Review articles on this cascade of insulin transduction pathways have been published [125,126]. As discussed above, metformin that modulates the AMPK pathway affects the translocation of GLUT4 in insulin target cells [127]. Hence, agents that facilitate the expression or mobilize GLUT4 could have antidiabetic effect. In this regard, the various effect of monoterpenes in signaling pathways appear to be consistent with antidiabetic effect through upregulation of GLUT4 expression and/or translocation (Table 1). The modulation of the AMPK pathway by natural products that enhance glucose uptake while concomitantly inhibiting gluconeogenesis and stimulation of glycogen synthesis have previously been reported (e.g., [128,129,130,131,132]. Hence, possible multiple mechanisms involving insulin signaling and other pathways involving the AMPK, GLUT4 and PPARγ are at play in the antidiabetic effect of monoterpenes.

The liver plays a central role in glucose homeostasis by regulating glucose production and storage in the form of glycogen. In terms of drug therapy, agents that suppress the glycolysis pathways (glycogenolysis inhibitors) or hepatic glucose production (gluconeogenesis) have antidiabetic properties [133,134,135,136]. These classical examples of antidiabetic (T2D) drugs therapy approaches target key enzymes involved in the cascades of reaction pathways in hepatic cells. The most important validated targets constitute the first important step of glycogenolysis that requires the enzymatic action of glycogen phosphorylase (GP) to convert glycogen to glucose-1-phosphate. On the other hand, glucose-6-phosphatase (G6Pase) at the final step of gluconeogenesis serves as an important drug target. In this regard, the effects of many monoterpenes as modulators of these pathways have been reported (Table 2). Hence, the antidiabetic mechanism of monoterpenes may include gluconeogenesis and glycolysis as potential targets.

The role of inflammation as a link between diabetes and obesity has been documented in recent years [137]. The low grade persistent/chronic state of inflammation in obesity is generally considered to increase insulin resistance in target organs [138]. The closer link between inflammation under obesity condition and insulin resistance in diabetes has become clearer with the identification of proinflammatory cytokines such as tumour necrosis factor (TNF)-α and interleukin-6 (IL-6) as major culprits [139,140]. For example, the above-mentioned PI3K-AKT (or PKB) pathway of the insulin-signaling cascade can be inhibited by these cytokines via activation of the NF-κB. This crosstalk between the inflammation pathway and obesity/diabetes as mechanisms of action for natural products such as anthocyanins has also been described recently [122]. As various monoterpenes appear to suppress the inflammation associated with diabetes (Table 1 and Table 2), their potential therapeutic effect could be in part attributed to such pharmacological effects. Hand in hand with all these multiple mechanism, the lipid lowering and/or antiobesity effect of monoterpenes appear to be demonstrated (Table 2). In this connection, the direct effect of monoterpenes on lipogenic expression via targeting key fatty acid synthesis enzymes (acetyl-CoA carboxylase and fatty acid synthase) have been demonstrated in vitro [42].

Besides the diverse possible mechanisms of actions that accounts to the pharmacological effects of monoterpenes, the reported specific effect through modulation of the GLP-1 receptor is very interesting. The best studied compound in this regard is the iridoid glycoside, geniposide [38]. By using the receptor antagonist, exenidin, the effect of geniposide through this mechanism has been confirmed [38,39].

In summary, in vitro and in vivo studies on the antidiabetic effects of monoterpenes so far have largely been demonstrated to show their promises as potential antidiabetic along with potential antiobesity and lipid lowering agents. One crucial evidence still missing is that on human/clinical studies. These compounds as structurally simple as they seem to be have good set of effects with a further opportunity to optimize their activity through structure-activity studies. Such an approach is also largely unexplored so far as these compounds appear to be relatively newcomers in antidiabetic study. The incorporation of these compounds with other structural groups such flavonoids [46] is an interesting development. As such, flavonoids have been demonstrated to have potent antioxidant effect that is of great interest in ameliorating both the diabetes-induced glycation and oxidative injuries. Flavonoids and other phenolic compounds are also known for their α-glucosidase inhibition and other mechanisms involved in diabetes pathology. The formulation of monoterpenes is also another avenue of development in their potential antidiabetic therapeutic effects. Their extreme non-polar nature, though often compensated by glycosylation, would need further studies in modifying their bioavailability and general pharmacokinetic profiles. In this direction, the potential of in silico studies in lead prediction and optimization should also be fully employed. Exemplary indexing model on filtering and mapping discriminative physicochemical properties for identifying antidiabetic natural products have also been published [141]. With all these developments awaiting, the current level of evidences suggest that monoterpenes indeed have a promise as antidiabetic lead compounds through multiple mechanisms as evidenced from both in vitro and in vivo studies.

## Figures and Tables

**Table 1:** In vitro antidiabetic effect of monoterpenes.

Compound	Model	Outcome	References
Carvacrol	H_2_O_2_-induced cellular injury on isolated pancreas islets—Following 20, 40 and 80 mg/kg/day in vivo treatment	Cytoprotective	[29]
Cymene	Advanced glycation end *products* (AGEs)	100 μM—Inhibit AGE formation; inhibit glycation specific decline in BSA α-helix content and β-sheet.	[30]
Genipin	C2C12 myotubes	10 μM—Stimulate glucose uptake; promote GLUT4 translocation; increase insulin receptor IRS-1, AKT, and GSK3β phosphorylation; increase ATP levels, close K(ATP) channels; increase intracellular calcium level; effect blocked by wortmannin and EGTA *.	[31]
Geniposide	Rat INS-1 pancreatic β cells	Prevent cell damage induced by high (25 mM) glucose through the AMPK pathway	[32,33]
Geniposide	Pancreatic β-cells—cultured primary cells of rats origin	10 µM—Potentiate insulin secretion via activating the glucagon-like-1 receptor (GLP-1R) as well as the adenylyl cyclase (AC)/cAMP signaling pathway; inhibit voltage-dependent potassium channels; activate Ca^2+^ channels.	[34]
Geniposide	Primary cortical neurons; PC12 cells	Enhance PPARγ phosphorylation; accelerate the release of phosphorylated FoxO1 (forkhead box O1) from nuclear fraction to the cytosol; activate the activity of insulin-degrading enzyme promoter in PC12 cells	[35]
Geniposide	INS-1 pancreatic β cells	10 μM—Increase phosphorylation of PDK1 and Akt473; inhibit the phosphorylation of downstream target GSK3β; increase expression of GLUT2; effect abolished by inhibitor of PI3K (LY294002).	[36]
Geniposide	INS-1 pancreatic β cells	Up to 10 μM—Enhance glucose-stimulated insulin secretion in response to low or moderately high glucose concentrations; promote glucose uptake and intracellular ATP levels; modulate pyruvate carboxylase expression.	[37]
Geniposide	Pancreatic INS-1 cells	Attenuate palmitate-induced β-cell apoptosis and caspase-3 expression; improve the impaired GLP-1R signaling by enhancing the phosphorylation of Akt and Foxo1; increase the expression of PDX-1; effect inhibited by exendin (9–39), an antagonist for GLP-1 receptor.	[38]
Geniposide	Pancreatic INS-1 cells	10 μmol/L—Enhance acute insulin secretion in response to both the low (5.5 mmol/L) and moderately high levels (11 mmol/L) of glucose; Effect inhibited by GLP-1R antagonist exendin (9–39) or knock-down of GLP-1R with shRNA interference in INS-1 cells.	[39]
Geniposide	HepG2 fatty liver model- free fatty acid treatment	Suppress the intracellular lipid accumulation; increase the intracellular expression of a fatty acid oxidation-related gene (PPARα).	[40]
Gentiopicroside	HL1C hepatoma cells	50 and 100 µM—Suppress Pck1 expression; induce phosphorylation of components in the insulin signaling cascade (Akt and Erk1/2 phosphorylation).	[41]
Paeoniflorin	3T3-L1 adipocytes treated with tumour necrosis factor (TNF)-α	50 µg/mL—Increase insulin-stimulated glucose; promote serine phosphorylation of IRS-1 and insulin-stimulated phosphorylation of AKT; inhibit the expressions and secretions of IL-6 and MCP-1; attenuate TNF-α-mediated suppression of the expressions of PPARγ and PPARγ target gene; effect reversed by antagonist of PPARγ activity.	[42]
Paeoniflorin	3T3-L1 adipocytes and RAW 264.7 macrophages	12.5–100 µg/mL—Inhibit TNF-α and FFA production; inhibit TNF-α-stimulated adipocyte lipolysis; suppress phosphorylation of TNF-α-activated ERK1/2; attenuate (partially) palmitate-induced macrophage TNF-α production.	[43]
Paeoniflorin derivatives (methoxyl and glucoside analogues)	Human HepG2 cells and HUVECs	10 µM—Increase glucose uptake; reverse glucose-induced inhibition of glycogen synthesis in HepG2; increase AMPK and GSK-3β phosphorylation; phosphorylate AMPK and increase phosphorylation of GSK-3β while suppressing lipogenic expression (acetyl-CoA carboxylase and fatty acid synthase); induced eNOS phosphorylation in HUVECs.	[44]
(R)-(+)-limonene	3T3-L1 cell culture; α-amylase and α-glucosidase enzymes	Increase GLUT1 expression at mRNA level; Weak enzyme inhibition (mM range).	[45]
Saturejin (3′-(2,5-dihydroxy-*p*-cymene) 5,7,4′-trihydroxy flavone) from *Satureja khuzistanica* Jamzad	Antioxidant activity; α- and β-glucosidase inhibitory	10 μg/mL—Significant in vitro radical (DPPH) scavenging and enzyme inhibitory effects.	[46]
Sweroside	HL1C hepatoma cells	Suppress Pck1 expression and induce phosphorylation of components in the insulin signaling cascade (Akt and Erk1/2 phosphorylation).	[41]
Swertiamarin	Steatosis in HepG2 cells induced by 1 mM oleic acid	25 μg/mL—Maintain membrane integrity; prevent apoptosis; increase the expressions of major insulin signaling proteins (insulin receptor, PI3K and pAkt) with concomitant reduction in p307 IRS-1; activate AMPK; modulate PPAR-α; decrease the levels of the gluconeogenic enzyme, PEPCK.	[47]
Thujone	Palmitate-induced insulin resistance in skeletal muscle (Soleus muscles)	Ameliorate palmitate oxidation and enhance insulin-stimulated glucose transport; restore (partially) GLUT4 translocation and AS160 phosphorylation; increase AMPK phosphorylation.	[48]

* EGTA represent ethylene glycol bis(2-aminoethyl ether)tetraacetic acid.

**Table 2:** In vivo antidiabetic effects of monoterpenes.

Compound	Model	Outcome	References
Aucubin	STZ-induced diabetic rats—5 mg/kg, i.p. twice daily for the first 5 days followed by single injections daily for 10 days.	Lower blood glucose; reverse lipid peroxidation and the decreased in activities of antioxidant enzymes in liver and kidneys; increase immunoreactive beta cells.	[66]
Borneol	STZ-induced diabetic rats—25 or 50 mg/kg, p.o. for 30 days.	Lower blood glucose and HbA1c; increase blood insulin; restore body weight loss; increase liver glycogen level; reverse the diabetes-induced increase in the levels of TC, TGs LDL-C, VLDL-C; restore urea and ALT and AST levels; increase antioxidant status (SOD, catalase, GSH) in the liver and kidney; reduce MDA level.	[67]
Carvacrol	HFD-induced C57BL/6J diabetic mice—20 mg/kg p.o. for 35 days.	Suppress elevated TC, TG, phospholipids and FFAs, VLDL-C, LDL-C in plasma and tissues; Suppress liver tissue inflammatory cytokines (TNF-α and IL-6); increase high density lipoproteins-cholesterol (HDL-C)	[68]
Carvacrol	HFD-induced type 2 diabetic C57BL/6J mice—20 mg/kg, p.o. for 35 days.	Ameliorate the increased glucose-6-phosphatase and fructose-1,6-bisphosphatase, decreased glucokinase and glucose-6-phosphate dehydrogenase activities; normalize hepatic markers (ASP, ALA, ALP, and γ-glutamyl transpeptidase).	[69]
Carvacrol	STZ-induced diabetic rats—25, 50, and 100 mg/kg, p.o. for 7 weeks or 20, 30 and 40 i.p.	Improve diabetes-associated cognitive deficit; suppress oxidative stress (increased MDA level and decreased SOD as well as reduced GSH) and inflammatory and apoptosis markers (NF-κB p65 unit, TNF-α, IL-1β, and caspase-3).	[70,71]
Carvacrol	STZ-induced diabetes in rats—25 and 50 mg/kg, p.o. for 7 days.	Suppress serum glucose, total cholesterol, ALA, AST and lactate dehydrogenase; no effect on serum insulin levels, food-water intake values and body weight changes.	[72]
Carvone	STZ-induced diabetic rats—50 mg/kg, p.o. for 30 days.	Reduce plasma glucose, HbA1c; improve the levels of hemoglobin and insulin. Revers activities of carbohydrate metabolic enzymes, enzymatic antioxidants and hepatic marker enzymes.	[73]
Catalpol	STZ-induced diabetic rats—10 mg/kg, i.p. for 14 days.	Improve impaired renal functions; ameliorate pathological changes in kidneys; abolish the diabetes induced elevation of Grb10 expression in the kidneys; increase IGF-1 mRNA levels and IGF-1R phosphorylation in kidneys.	[74]
Catalpol	HFD-fed mice receiving 100 mg/kg, p.o. for 4 weeks	No effect on body weight; improve fasting glucose and insulin levels, glucose tolerance and insulin tolerance; reduce macrophage infiltration into adipose tissue; reduce mRNA expressions of M1 pro-inflammatory cytokines while increasing M2 anti-inflammatory gene expressions in adipose tissue; suppress the JNK and NF-κB signaling pathways in adipose tissue.	[75]
Catalpol	STZ-diabetic rats—0.1 mg/kg, i.p.	Enhance glucose uptake in the isolated soleus muscle of diabetic rats; increase glycogen synthesis.	[76]
Catalpol	STZ-induced diabetic rats—10, 50 and 100 mg/kg, p.o. for 6 weeks.	Improve neuronal injury and cognitive dysfunction; increase the nerve growth factor concentration and decrease the blood glucose.	[77]
Citronellol	STZ-induced diabetic rats—25, 50, and 100 mg/kg, p.o. for 30 days.	Improve the levels of insulin, hemoglobin and hepatic glycogen with significant decrease in glucose and HbA1c levels. Restore altered activities of carbohydrate metabolic enzymes and level of hepatic and kidney markers; improve morphology of hepatic cells and insulin-positive β-cells.	[78]
Cymene	STZ-induced diabetic rats—20 mg/kg, p.o. for 60 days	Improve HbA1c and nephropathic parameters (like albumin excretion rate, serum creatinine and creatinine clearance rate).	[30]
Genipin	Aging rats—25 mg/kg, i.p. for 12 days	Ameliorate systemic and hepatic insulin resistance, alleviate hyperinsulinemia, hyperglyceridemia and hepatic steatosis, relieve hepatic oxidative stress and mitochondrial dysfunction; improve insulin sensitivity by promoting insulin-stimulated glucose consumption and glycogen synthesis; inhibit cellular ROS overproduction and alleviate the reduction of levels of MMP and ATP.	[79]
Geniposide	Insulin-deficient—APP/PS1 transgenic mouse model of Alzheimer’s disease. 5, 10, and 20 mg/kg, intragastric for 4 weeks.	Decrease the phosphorylation of tau protein.	[80]
Geniposide	STZ-induced diabetic rats—injection (50 μM, 10 μL) to the lateral ventricle	Prevent spatial learning deficit; reduce tau phosphorylation.	[81]
Geniposide	Transgenic mouse model with Streptozotocin—5, 10 and 20 mg/kg, intragastric for 4 weeks	Decreased level of β-amyloid peptides (Aβ1-40 and Aβ1-42); up-regulate the protein levels of β-site APP cleaving enzyme (BACE1) and insulin-degrading enzyme (IDE); decrease the protein levels of ADAM10; enhance the effects of insulin by reducing Aβ1-42 levels in primary cultured cortical neurons.	[35]
Geniposide	STZ-induced diabetic rats—800 mg/kg/day, p.o. for 46 days.	Improve insulin and blood glucose; decrease Aβ1-42 level; improve the expression of insulin-degrading enzyme.	[82]
Geniposide	Spontaneously obese Type 2 diabetic TSOD mice	Suppress body weight, visceral fat and intrahepatic lipid accumulation; alleviate abnormal lipid metabolism; alleviate abnormal glucose tolerance and hyperinsulinemia.	[40]
Geniposide	High fat diet—25, 50 or 100 mg/kg, p.o. for six weeks.	Improve liver histology through reducing the elevated liver index (liver weight/body weight), serum alanine aminotransferase and aspartate aminotransferase; decrease total cholesterol, triglycerides and FFAs in serum and liver; increased serum insulin levels but reduced serum TNF-α level; suppressed expression of CYP2E1 and increased PPARα expression	[83]
Geniposide	HFD and STZ-induced diabetic mice −200 and 400 mg/kg for 2 weeks	Decrease blood glucose, insulin and TG levels; decrease the expression of glycogen phosphorylase and glucose-6-phosphatase at mRNA level and immunoreactive protein levels, as well as enzyme activity.	[84]
Geraniol	STZ-induced diabetic rats—100, 200 and 400 mg/kg, p.o. for 45 days	Improve the levels of insulin, hemoglobin and decrease plasma glucose, HbA1c; improve hepatic glycogen content; preserve the normal histological appearance of hepatic cells and pancreatic β-cells.	[85]
d-Limonene	STZ-induced diabetic rats—50, 100 and 200 mg/kg, p.o. for 45 days.	Reverse the following diabetic effect: increased blood glucose and glycosylated hemoglobin levels, increased activity of gluconeogenic enzymes (glucose 6-phosphatase and fructose 1,6-bisphosphatase) and decreased activity of glycolytic enzyme, glucokinase and liver glycogen.	[86]
d-Limonene	STZ-induced diabetic rats—50 mg/kg, p.o. for 28 days.	Decrease DNA damage, glutathione reductase enzyme activities and MDA levels; increase GSH levels and CAT, SOD and GSH-Px enzyme activities and altered lipid and liver enzyme parameters in diabetic rats.	[87]
Logonin	STZ-induced diabetic mice, 20 mg/kg, p.o. for 12 weeks.	Reduce kidney/body weight ratio, 24 h urine protein levels, serum levels of urea nitrogen and creatinine; improve histology of pancreas and kidney; alleviate structural alterations in endothelial cells, mesangial cells and podocytes in renal cortex; reduce AGE levels in serum and kidney; downregulate mRNA and protein expression of receptors for AGEs in kidney; reduce the levels of MDA; increase the levels of SOD in serum and kidney.	[88]
Menthol	STZ-nicotinamide induced diabetes in rats—25, 50, and 100 mg/kg, p.o. for 45 days	Reduce blood glucose and glycosylated hemoglobin levels; increase the total hemoglobin, plasma insulin and liver glycogen levels; protect hepatic and pancreatic islets; modulating glucose metabolizing enzymes, suppression of pancreatic β-cells apoptosis and altered hepatic, pancreatic morphology	[89]
Myrtenal	STZ-induced diabetic rats—80 mg/kg, p.o. for 28 days	Decrease plasma glucose; increase plasma insulin levels; up-regulate IRS2, Akt and GLUT2 in liver; increase IRS2, Akt and GLUT4 protein expression in skeletal muscle.	[90]
Myrtenal	STZ-induced diabetic rats—20, 40, and 80 mg/kg, p.o. for 28 days	Reduce plasma glucose, haemoglobin A1c (HbA1c); increase the levels of insulin and hemoglobin; reverse body weight loss; normalize hexokinase, glucose-6-phosphatase, fructose-1,6-bisphosphatase, glucose-6-phosphate dehydrogenase, and hepatic enzymes AST, ALT, and ALP levels; improve hepatic and muscle glycogen content; restore islet cells and liver histology.	[91]
Myrtenal	STZ-induced diabetic rats—80 mg/kg, p.o. for 28 days	Improve plasma glucose, pancreatic insulin and lipid profiles (TC, TG, FFAs, phospholipids, LDL, VLDL, atherogenic index); improve histopathological feature of the liver.	[92]
Paeoniflorin	High-sucrose, HFD rat receiving low dose STZ—15 and 30 mg/kg, p.o. for 4 weeks.	Reduce brain inflammatory cytokines (IL-1β and TNF-α), decrease suppressor of cytokine signaling 2 expressions and promote (IRS-1 activity and phosphorylation levels of protein kinase B (Akt) and glycogen synthase kinase-3β (GSK-3β).	[93]
Paeoniflorin	HFD—induced obese mice—Diet containing 0.05% (*w*/*w*) of paeoniflorin	Lower body weight, hyperlipidemia, and insulin resistance; block inflammation; inhibiting lipid ectopic deposition; lower lipid synthesis pathway (de novo pathway, ^3^HMG-CoAR), promote fatty acid oxidation (peroxisome proliferator-activated receptor-alpha (PPARα), carnitine palmitoyltransferase-1); increase cholesterol output (PPARγ-liver X receptor-α-ATP-binding cassette transporter-1); block inflammatory genes activation and reduce gluconeogenic genes expression (phosphoenolpyruvate carboxykinase and G6Pase).	[82]
Swertiamarin	STZ-induced diabetic rats—50 mg/kg, i.p. for 6 weeks	Reduce serum triglycerides, cholesterol and low-density lipoprotein levels; decrease serum fasting glucose; increase insulin sensitivity index.	[94]
Thymol	HFD-induced type 2 diabetes in C57BL/6J mice—10, 20 and 40 mg/kg, intragastric for 5 weeks.	Antihyperglycaemic; lower plasma TG, TC, FFAs, LDL and increase HDL cholesterol; lower hepatic lipid contents TG, total cholesterol, FFAs and phospholipids.	[95]
Thymol	HFD- induced type 2 diabetes in C57BL/6J mice—40 mg/kg, intragastric for 5 weeks.	Inhibit diabetic nephropathy; inhibit the activation of transforming growth factor-β1 and vascular endothelial growth factor; increase level of antioxidants and suppress lipid peroxidation markers in erythrocytes and kidney tissues; downregulate the expression level of sterol regulatory element binding protein-1c and reduce lipid accumulation in the kidney.	[96]
Thymol	HFD-induced obese C57BL/6 J mice—20, 40 mg/kg daily	Reverse body weight gain; ameliorate peripheral insulin resistance; improve cognitive impairments in the Morris Water Maze test; decrease HFD-induced Aβ deposition and tau hyperphosphorylation in the hippocampus; down-regulate the level of P-Ser307 IRS-1 and enhance the expression of P-Ser473 AKT and P-Ser9 GSK3β; up-regulate nuclear respiratory factor /heme oxygenase-1pathway.	[97]
Thymol	HFD-fed rats—14 mg/kg, p.o. for 4 weeks.	Decrease body weight gain, visceral fat -pad weights, lipids, ALT, AST, LDH, blood urea nitrogen, glucose, insulin, and leptin levels; decrease serum lipid peroxidation and increase antioxidant levels.	[98]

## Abbreviations

Aβ	Amyloid Beta
AMPK	Adenosine Monophosphate-Activated Protein Kinase
AKT	Protein Kinase B (PKB)
AGE	Advanced Glycation End Products
ALP	Alkaline Phosphatase
ALT	Alanine Transaminase
AST	Aspartate Aminotransferase
BACE1	Beta-Secretase 1
CAT	Catalase
CYP2E1	Cytochrome P450 2E1
DMAPP	Dimethylallyl Diphosphate
DPPH	2,2-Diphenyl-1-Picrylhydrazyl
EGTA	Ethylene Glycol-bis(β-Aminoethyl Ether)-*N*,*N*,*N*′,*N*′-Tetraacetic Acid
Erk	Extracellular Signal–Regulated Kinases
HFD	High Fat Diet
HDL	High Density Lipoproteins
HMG-CoAR	3-Hydroxy-3-Methyl Glutaryl Coenzyme A Reductase
FFAs	Free fatty acids
FoxO1	Forkhead Box O1
GLP-1R	Glucagon-Like-1 Receptor
GLUT	Glucose Transporter
GPx	Glutathione Peroxidase
Grb10	Growth Factor Receptor Bound Protein 10
GSH	Glutathione—Reduced Form
GSK3β	Glycogen Synthase Kinase 3 Beta
HbA1c	Haemoglobin A1c
HUVECs	Human Umbilical Vein Endothelial Cells
IDE	Insulin-Degrading Enzyme
IGF-1	Insulin-Like Growth Factor-1
IRS	Insulin Receptor Substrate
IPP	Isopentenyl Diphosphate
IL	Interleukin
JNK	c-Jun N-terminal kinase
LDH	Lactate Dehydrogenase
LDL	Low Density Lipoproteins-Cholesterol
MCP-1	Monocyte Chemoattractant Protein-1
MDA	Malondialdehyde
MMP	Mitochondrial Membrane Potential
NF-κB	Nuclear Factor Kappa-Light-Chain-Enhancer of Activated B Cells
Pck1	Phosphoenolpyruvate Carboxykinase 1
PI3K	Phosphatidylinositide 3-Kinase
PDK1	3-Phosphoinositide-Dependent Protein Kinase-1
PDX-1	Pancreatic and Duodenal Homeobox 1 or Insulin Promoter Factor 1
PEPCK	Phosphoenolpyruvate Carboxykinase
PPAR	Peroxisome Proliferator-Activated Receptor
ROS	Reactive Oxygen Species
SOD	Superoxide Dismutase
T1D	Type-1 Diabetes
T2D	Type-2 Diabetes
TC	Total Cholesterol
TG	Triglycerides
TNF	Tumor Necrosis Factor
VLDL	Very Low Density Lipoproteins
